# Supplementary material for: Utilization of maternal waiting home and associated factors among women who gave birth in the last one year, Dabat district, Northwest Ethiopia
Source: PLoS One. 2022 Jul 8;17(7):e0271113. doi: 10.1371/journal.pone.0271113 (PMC9269408; doi:10.1371/journal.pone.0271113)
Supplement: S1 File — (DOCX) [file pone.0271113.s001.docx]

Questionnaire

English version questionnaire

| 1. Socio-demographic information | | | |
| --- | --- | --- | --- |
| S.no | Question | Response | Comments |
| 101 | How old are you ? | Age in years__________ |  |
| 102 | What is Your Marital status? | 1. Single 2. Married 3. divorced 4. Widowed |  |
| 103 | What is Your religion ? | 1. Orthodox 2. Muslim 3. protestant 4. Others (specify)_____ |  |
| 104 | What is Your Level of education? | 1. No formal education 2. Primary 3. Secondary 4. Vocational 5. Diploma 6. Degree and above |  |
| 105 | What is Your Occupational status? | 1. Merchant 2. Farming 3. Employee 4. Others(specify)___ |  |
| 106 | What is Your Partner educational status? | 1. No formal education 2. Primary 3. Secondary 4. Vocational 5. Diploma 6. Degree and higher |  |
| 107 | How much time takes from your house to the nearest Health Center | ­­­­­­­­­­­___________Minutes |  |
| 108 | Accessibility of transport at the time of child birth related emergency | 1. Easy to get 2. Hard to get |  |
| 109 | Can you afford transport costs at the time of child birth related emergency, if there is no ambulance or delay? | 1. Affordable 2. Not affordable |  |
| 110 | If child birth related emergency happened, what kind of means of transport you will use longer distances to nearest health center | 1. Traditional ambulance 2. By horse back 3. By car 4. By ambulances 5. If others (specify)_____________ |  |

| 1. Reproductive and obstetric related factors | | | |
| --- | --- | --- | --- |
| s.no | Question | Response | Comments |
| 201 | Who usually decide on your health | 1. Respondent 2. Husband 3. Jointly 4. Others (specify)______ |  |
| 202 | How often did you give birth to a live baby? | _______________ |  |
| 203 | Total no of pregnancies ? | _________________ |  |
| 204 | Do you have history of IUFD/still births? | 1. Yes 2. No |  |
| 205 | How many ANC visits did you have in your last pregnancy? | ___________ |  |
| 206 | Was planned of your last pregnancy? | 1. Planned 2. Un planned |  |
| 207 | Did you know your expected date of delivery during your pregnancy? | 1. Yes 2. No |  |
| 208 | Do you received information about danger signs during pregnancy? | 1. Yes 2. No |  |
| 209 | Can you tell me any danger signs during pregnancy? | 1. Vaginal bleeding 2. Vaginal flush of fluid 3. Severe headache 4. Blurred vision 5. Severe abdominal pain 6. Fever 7. Decreased fetal movement 8. Edema or body swelling 9. Fainting 10. Others (specify)__________ |  |
| 210 | Do you received information about birth preparedness plan during ANC visits | 1. Yes 2. No |  |
| 211 | Birth place of the current child? | 1. Home 2. Health institution |  |

| 1. Information’s about MWHs | | | |
| --- | --- | --- | --- |
| s.no | Question and filters | Response | Comments |
| 301 | Have you ever heard of a Maternity Waiting Home? | 1. Yes 2. No |  |
| 302 | Do you know Where is the MWH located in your cluster | 1. Yes 2. No |  |
| 1. Utilization of Maternity waiting home services | | | |
| 401 | Did you stay at a Maternity Waiting Home in last pregnancy? | 1. Yes 2. No |  |
| 402 | If yes how many days? | __________________ |  |

| 1. Social and behavioral factors | | | | |
| --- | --- | --- | --- | --- |
| 501 | Did you perceive the specified time (Staying for 2-4 weeks) before labor when to wait /while waited in MWHs is acceptable? | 1. Acceptable 2. Not Acceptable |  |  |
| 502 | Possibility of getting Attendant when to wait /while waited in MWHs | 1. Possible 2. Not possible |  |  |
| 503 | Possibility of getting people (care giver) for your children when to wait /while waited in MWHs | 1. Possible 2. Not possible |  |  |
| 504 | Possibility of getting people for Household chores when to wait /while waited in MWHs | 1. Possible 2. Not possible |  |  |

**Amharic version questionnaire**

| 1. ማህበራዊ ሁኔታ | | | |
| --- | --- | --- | --- |
| s.no | ጥያቄ | አማራጭ/መልስ መስጫ |  |
| 101 | የትውልድዘመን/እድሜ? | በአመት___________________ |  |
| 103 | የትዳርሁኔታ ? | 1. ያገባች 2. ያላገባች 3. የተፋታች 4. የሞተባት |  |
| 103 | የሚከተሉትሃይማኖት? | 1. ኦርቶዶክስ 2. ሙስሊም 3. ፐሮቴስታንት 4. ሌላካለይገለጽ________________ |  |
| 104 | የትምህርት ደረጃ | 1. መደበኛት/ትየለላቸዉ 2. የመጀመሪያደረጃ 3. ሁለተኛደረጃ 4. ሙያናቴክኒክ 5. ዲፕሎማ 6. ዲግሪእናከዚያበላይ |  |
| 105 | የ ርስዎ ስራ ምንድንነዉ | 1. ነጋዴ 2. ግብርና 3. ተቀጥሬ (በመንግስት/በግል) 4. ሌላ ካለ ይጠቀስ____________ |  |
| 106 | የትዳር ጎደኛዎ የትምህርት ደረጃ | 1. መደበኛ ት/ት የለላቸዉ 2. 1ኛደረጃ 3. ሁለተኛ ደረጃ 4. ሙያ ና ቴክኒክ 5. ዲፕሎማ 6. ዲግሪ ና ከዚያ በላይ |  |
| 107 | ከቤትዎት እስከ ባቅራቢያዎ ወደሚገኝ ጤና-ጣቢያ ለመጓዝ ምንያክል ጊዜ ይፈጃል? | _______________________ደቂቃ |  |
| 108 | ምጥ ወይም ከወሊድ ጋር ተያያዥችግር ቢገጥመዎ ትራንስ ፖርት ማግኘት ምንያክል ከባድ ወይም ቀላልነው | 1. ማግኘት ከባድ ነዉ 2. ማግኘት ቀላል ነዉ |  |
| 109 | ምጥ ወይም ከወሊድጋርተያያዥችግር ቢገጥመዎት እና አምቡላንስ በይኖር በግል ለትራንስፖርት ከፈለዉ ወደ ጤና ጣቢያ/ሆስፒታል ለመምጣት አቅመዎ ይፈቅዳል | 1. አቅሜ ይፈቅዳል 2. አቅሜ አይፈቅድም |  |
| 110 | ቤት በሚወልዱበት ወቅትችግር ቢፈጠር ጤና-ተቋም ለመድረስ አብዛኛውን መንገድ በምን ይጓዛሉ | 1. በቃሬዛ 2. በበቅሎ/ፈረስ 3. በመኪና 4. በአምቡላንስ |  |

| 1. **ስነተዋልዶ ጤናን በተመለከተ** | | | | | |
| --- | --- | --- | --- | --- | --- |
| s.no | ጥያቄ | አማራጭ |  | | |
| 201 | የእርሶዎን እና የልጅዎን ጤንነት በተመለከተ አብዛኛውን ጊዜ ማን ነው የሚወስነው? | 1. እኔ ራሴ 2. ባለቤቴ 3. በእኔ 4. ባለቤቴ 5. በጋራ 6. ሌላ ካለ ይጠቀስ________ |  | | |
| 202 | በሕይወትየተወለዱስንትልጆችአሉዎት? | ________________________ |  | |  |
| 203 | በጠቅላላ ስንት ጊዜ አርግዘዋል? | __________________________ |  | |  |
| 204 | ሕይወት የሌለው ልጅወልደ ውያው ቃሉ? | 1. አዎ 2. የለም |  | |  |
| 205 | በመጨረሻው እርግዝና ወቅት የህክምና ክትትል ስንት ጊዜ አድርገዋል? | _______________ |  | |  |
| 206 | እርግዝናው የታቀደበት ነበር? | 1. የታቀደ 2. ያልታቅደ |  | |  |
| 207 | የሚዎልዱበትን ቀን ያውቁ ነበር? | 1. አዎ 2. የለም |  | |  |
| 208 | በእርግዝናወቅት የህክምናክትትል በሚያደርጉበት ጌዜ ከእርግዝና ጋርተያያዥ ሰለሆኑ ችግሮች/ሊከሰቱ ሰለሚችሉ አደገኛ ምልክቶች ተነግሮወታል? | 1. አዎ 2. የለም |  | |  |
| 209 | እባክዎን በእርግዝና ጊዜ ሊከሰቱ የሚችሉ አደገኛ ምልክት ይዘርዝሩልኝ | 1. የደምመፍሰስ 2. ከምጥበፊትሽርትዉሃመፍሰስ 3. ዕይታብዥማለት 4. ከፍተኛሆድቁርጠት 5. የሰዉነትሙቀትመጨመር 6. የጽንሱእንቅስቃሴመቀነስ/ማቆም 7. ከፍተኛራስምታት 8. የሰዉነትማበጥ 9. ራስመሳት 10. ሌላከለይጠቀስ_____________ | |  | |
| 210 | በእርግዝናወቅት የህክምናክትትል በሚያደርጉበት ጊዜ ከወሊድ በፊት ማድረግ ስላለብዎት ቅድመዝግጅት መረጃ አግኝተዋል?  ግልፅካልሆነአስረዳ:ከወሊድበፊትሊደረግየሚገባቅድመዝግጅትስባልየሚከተሉትንያካትታል።  1.በእርግዝናወቅትበቅድሚያየመውለጃቦታንጤናተቋምከሆነችግርእንኩዋንቢፈጠርበኦፕሬሽንማዋለድየሚችልተቋምለይቶማዘጋጀት  2. በምንአይነትመጓጓዣወደጤናተቋሙመሄድእንዳለብዎ፤ለትራንሰፖርትናለያጋጥሙለሚችሉየህክምናወጭዎችሊከፈልየሚችልብርመቆጠብ  3.ብዙደምመፍሰስቢያጋጥምዎያንንለመተካትደምየሚለግስሰውቀድሞማዘጋጀት | 1. አዎ 2. የለም | |  | |
| 211 | የአሁኑን ልጅዎን የትነበር የወለዱት | 1. ቤት 2. ጤና ተቋም | |  | |

| 1. በእናቶች ማቆያ ዙሪያ ያለ መረጃ | | | | |
| --- | --- | --- | --- | --- |
| s.no | ጥያቄ | | አማራጭ |  |
| 301 | ሰለነፈሰጡር እናቶች ማቆያ ቤት ሰምተዉ ያዉቃሉ? | | 1. አዎ 2. የለም |  |
| 302 | የነፈሰጡር እናቶች ማቆያ ቤት የት እንዳለ ያዉቃሉ | 1. አዎ 2. የለም | |  |
| 4. የነፈሰጡር እናቶች ማቆያ ቤት ስለመጠቀም | | | | |
| 401 | በዚህ እርግዝና ነፈሰጡር እናቶች ማቆያ ቤት ተጠቅመዉ ያዉቃሉ? | 1. አዎ 2. የለም | |  |
| 402 | አዎ ካሉ ለምን ያክል ቀን | \| ________________________ቀን \| \| --- \| | |  |

| 5 ማህበራዊ እና ስነ ልቦናዊ ጉዳዮች | | | |
| --- | --- | --- | --- |
| 501 | የእናቶችማቆያ ሲቆዩ ወይም በነፈሰጡር እናቶች ማቆያ ቤት ለመቆየት ከመውለጃዎ ጊዜ ከ2 - 4 ሳምንት ቀደምብለዉ መቆየት አለብዎ ቢባሉ ይቀበሉታል? | 1. አወ እቀበለዋለሁ 2. ይከብደኛል |  |
| 502 | የእናቶች ማቆያ ሲቆዩ ወይም በነፈሰጡር እናቶች ማቆያ ቤት ለመቆየት አስታማሚ ማግኘት ይችሉ ነበር | 1. ቀላልነበር/ቀላልነዉ 2. ከባድነበር/ ይከብደኛ |  |
| 503 | የእናቶች ማቆያ ሲቆዩ ወይም በነፈሰጡር እናቶች ማቆያ ቤት ለመቆየት ልጆችዎን ተንከባካቢ ማግኘት ይችሉ ነበር | 1. ቀላልነበር/ቀላልነዉ 2. ከባድነበር/ ይከብደኛል |  |
| 504 | የእናቶች ማቆያ ሲቆዩ ወይም በነፈሰጡር እናቶች ማቆያ ቤት ለመቆየት የቤትዉስጥ ስራዉን የሚሰራ ማግኘት ይችሉ ነበር | 1. ቀላልነበር/ቀላልነዉ 2. ከባድነበር/ ይከብደኛል |  |
