# Supplementary material for: Utilization of maternal waiting home and associated factors among women who gave birth in the last one year, Dabat district, Northwest Ethiopia
Source: PLoS One. 2022 Jul 8;17(7):e0271113. doi: 10.1371/journal.pone.0271113 (PMC9269408; doi:10.1371/journal.pone.0271113)
Supplement: Attachment — Submitted filename: Response To Reviwor.docx [file pone.0271113.s003.docx]

PONE-D-21-25855

Response to editor and reviewers

Title: Utilization of maternal waiting home and associated factors among women who gave birth in the last one year, Dabat district, Northwest Ethiopia

First, the authors would like to thank Plos one Editor for grammar and editorial correction and the respected reviewers for reviewing our manuscript and providing the necessary comments to improve. The authors have made corrections point by point to the comments given and have tried to Responded all the issues raised by the editor and reviewers.

Response to editor comments

| S.N. | Comments | Response |
| --- | --- | --- |
| 1. | The topic is quite relevant towards reducing the burden of maternal and perinatal morbidity and mortality in the region. The manuscript however has a lot of grammatical errors which needs to be corrected | Thank you very much for the comments ,We agreed that the manuscript needs revision for grammar correction and it has been going through language revision |
| 2. | The justification for the study has not been clearly written. Authors needs to clearly justify why this study is relevant in the region, especially when there are several studies relating to utilization of maternity waiting homes in Ethiopia. | Thank you dear editor, we tried to justify the need of this study in the last paragraph of the introduction part. |
| 3. | The methodology is appropriate, but authors may need to justify the selection of variables with p-value of less than 0.2 instead of 0.05 for inclusion into multivariate logistic regression analysis | Thank you dear editor for your concern. We did based on the assumption to include all variables having a p-value of <0.25 as evidenced by existing literature (<http://dx.doi.org/10.21037/atm.2016.02.15>, doi: 10.1186/1751-0473-3-17, doi: 10.1186/1751-0473-3-17). For this study, we used a p-value of 0.25 assumption to proceed to the multivariable logistic regression, however, all variables which fulfill the chi-square assumption had a p-value of ≤ 0.2. If we include variable having a p-value of <0.05 to the multivariable analysis, we may totally miss important variables. Lastly, in the multivariable logistic regression, a p-value of 0.05 was used to declare the statistical association. |
| 4 | The result section needs to be revised with absolute values written before the percentage in brackets | Thank you, dear editor, for your insightful comments  we tried to correct as suggested |
| 5. | The authors have not discussed the findings with reference to the main objectives of the study rather there are repetitions of the results in several aspects of the discussion. The grammatical errors also made the flow cumbersome. The strengths and limitations of the study should be clearly highlighted | Thank you so much for your constructive suggestion  -we tried to revise the discussion and incorporated the strength and limitation at the revised version of the manuscript |
| 6 | Please also ensure that the references are as per journal recommendations(Vancouver referencing style)as many of the quoted references do not conform with this style. | Thank you very much for the comments, we have revised the references as per your kind recommendation |
| 7. | Why should a woman who mentions three out of nine listed danger signs be termed as knowledgeable? In all forms of assessment 33% cannot be termed as knowledgeable | Thank you very much for the comments   - We have taken this measurement by reviewing the literature (reference numbers 23-25). However, there is a lack of universal consensus in defining it. Some scholars consider it knowledgeable if the mother mentions two or above, some other three or above. |
| 8 | Other specific comments the editor indicated by item | Thank you, dear editor, for your insightful comments and deep review. We really appreciate your viewpoint. We tried to incorporate all of your comments in the revised manuscript |

Response to reviewers

Reviewer #1.

| S.N. | Comments | Response |
| --- | --- | --- |
| 1 | The manuscript needs a language editor | Thank you very much for the comments ,We agreed that the manuscript needs revision for grammar correction and it has been going through language revision |
| 2. | What are the inclusion and exclusion criteria? what are the outcome measures? | Thank you so much for your comment,  -the inclusion criteria for this study was all women who gave birth in the last one year prior to the study and living in the district  - Even though we didn’t get any participant in the actual data collection, seriously ill women were the exclusion criteria |
| 3. | INTRODUCTION  This is well written. However, the authors should beef up the justifications for the study. | Thank you dear reviewer, we tried to justify the need of this study in the last paragraph of the introduction part. |
| 4 | METHODS  There are so many typographical errors.  Eg:' Sample size determination and sapling procedure '. It is supposed to be sampling. | Thank you very much for the comments  - we tried to correct as suggested |
| 5. | RESULTS  The authors should begin with stating how many were assessed for eligibility and how many were excluded with reasons.  The authors should include a flowchart to illustrate the participants flow. | Thank you very much  -We took your comments, However, we have gotten 100% response rate or there is no excluded case. We tried to show the participants flow by flow chart (figer1) |
| 6. | What are the strengths and limitations | Thank you very much  We tried to incorporate in the revised manuscript |
|  |  |  |

Reviewer #2

| S.N | Comments | Response |
| --- | --- | --- |
| 1. | As this was a population based study the findings could be generalizable. However, it is not clear how household or individuals were selected. Did you do cluster sampling? in stages or what? Please in a sentence address your sampling procedure clearly. | Thank you dear reviewer for your concern   - We had been applied simple random sampling technique to select the study participants and we tried to make clear the sampling procedure in the revised manuscript (figure 1) |
| 2. | There a countless grammatical/spelling errors in both heading and texts. Sampling is typed as Sanpling; population as pollution, etc. | Thank you very much for the comments  - we tried to correct as suggested |
| 3. | The discussion has not been robust. Authors should restate their main objective in this discussion section and attempt addressing same | Thank you very much for your constructive comments  -We tried to revise and address |
| 4. | There is no clear cut strength and limitation of this study. | Thank you very much  We tried to incorporated in the revised manuscript |
