# Supplementary material for: Utilization of maternal waiting home and associated factors among women who gave birth in the last one year, Dabat district, Northwest Ethiopia
Source: PLoS One. 2022 Jul 8;17(7):e0271113. doi: 10.1371/journal.pone.0271113 (PMC9269408; doi:10.1371/journal.pone.0271113)
Supplement: Attachment — Submitted filename: response to reviwer.docx [file pone.0271113.s004.docx]

PONE-D-21-25855R1

Response to reviewer

Title: Utilization of maternal waiting home and associated factors among women who gave birth in the last one year, Dabat district, Northwest Ethiopia

First, the authors would like to thank Plos one Editor for grammar and editorial correction and the respected reviewers for reviewing our manuscript and providing the necessary comments to improve. The authors have made corrections point by point to the comments given and have tried to Responded all the issues raised by the editor and reviewers.

Response to editor comments

| S.N. | Comment | Response |
| --- | --- | --- |
| 1 | The manuscript has extensively been revised but there are still some typographical and grammatical mistakes which needs to be corrected | Thank you very much, dear editor.  we author tried to correct the typographic and grammatical mistakes. |
| 2. | Additionally, the following observed mistakes should be corrected during the revision of this manuscript. | Thank you in advance, dear editor; we accept your suggestion and tried to correct it. |

Response to reviewer 1

| S.N. | Comment | Response |
| --- | --- | --- |
|  | The authors have responded adequately. They have corrected all the typographical errors. There is need to address all issues raised | Thank you very much, dear reviewer, for your constrictive comments and acceptance of our revision. |

Response to reviewer 2

| S.N. | Comment | Response |
| --- | --- | --- |
| 1. | The study is interesting and present a great strategy for improvement of maternal and newborn health. There is however some concerns. The study design has not been stated and rationale for sample size has not been described. The discussion has not been robust as literature reviews have been sparse and uncoordinated. Authors have also not stated strength and limitation of their study. | Thank you very much, dear reviewer.  We had tried to state clearly our study design.  We also tried to use our efforts to make robust the discussion in our previous revision. Furthermore, we tried to add the limitations and strength of our study. |
| 2. | Gramma errors such as pollution instead of population, sapling instead of sampling, etc need to be corrected. | Thank you, dear editor,  We had already corrected this grammatical error in the previous revision. |
